# Spencer Wells’ Ovariotomies

**Published:** 1878-10

**Authors:** 


Spencer Wells' Ovariotomies.—Dr. Spencer Wells has performed 900 operations for ovariotomy. Of these there have been 676 total recoveries. Dr. Wm. Farr calculated that the expectation of life gained by these operations was in all 19,691 years.—*New York City Hospital Gazette.*

